# Relationship between parents’ health literacy and children’s sleep problems in Japan

**DOI:** 10.1186/s12889-021-10864-z

**Published:** 2021-04-24

**Authors:** Sae Ono, Hiroto Ogi, Masato Ogawa, Daisuke Nakamura, Teruhiko Nakamura, Kazuhiro P. Izawa

**Affiliations:** 1 Department of Physical Therapy, Faculty of Health Sciences, Kobe University School of Medicine, 7-10-2 Tomogaoka, Suma-ku, Kobe, 654-0142 Japan; 2 Cardiovascular Stroke Renal Project (CRP), 7-10-2 Tomogaoka, Suma-ku, Kobe, 654-0142 Japan; 3 Department of Public Health, Graduate School of Health Sciences, Kobe University, 7-10-2 Tomogaoka, Suma-ku, Kobe, 654-0142 Japan; 4 Division of Rehabilitation Medicine, Kobe University Hospital, 7-5-2 Kusunoki-cho, Chuo-ku, Kobe, 650-0017 Japan; 5 Fudousan Technologies Corporation, 1-29-2 Yurinokidai, Sanda, 669-1324 Japan; 6 Educational Corporation Tsukushi Gakuen, 2-3-11 Takadai, Chitose, 066-0035 Japan

**Keywords:** Parents, Health literacy, Children, Sleep problems, Preschoolers

## Abstract

**Background:**

Sleep problems in preschool children can stunt their health and growth. However, the factors that cause sleep problems in children are not well understood. The aim of this study was to determine the relationship between parents’ health literacy (HL) and children’s sleep problems. The study was conducted at two kindergartens, two nursery schools, and a center for early childhood education in Chitose-city, Hokkaido, Japan.

**Method:**

This study used a multicenter cross-sectional design. The sample comprised 354 preschoolers (aged 3–6 years) and their parents. In families with two or more children attending the same facility, only the oldest child was asked to participate in the study. Exclusion criteria included participants whose completed questionnaires had missing values. Children’s sleep problems were assessed using the Japanese version of the Children’s Sleep Habits Questionnaire (CSHQ-J). Parents’ HL was assessed using the 14-item Health Literacy Scale (HLS-14). The parents were classified into two groups (high HL group and low HL group). Multiple regression modelling was used to determine the association between HLS-14 and CSHQ-J scores.

**Results:**

Of the 354 parents, 255 (72%) were in the high HL group and 99 (28%) in the low HL group. The mean CSHQ-J score was significantly lower in the high HL group than in the low HL group (45.3 ± 6.0 points vs. 46.8 ± 5.9 points, *p* = 0.043). In multiple regression analyses, parents’ HL was independently associated with their CSHQ-J score after adjusting for all confounding factors (adjusted *R*^*2*^ = 0.22, β = − 0.11; *p* = 0.043).

**Conclusions:**

Parents’ HL appears to affect their children’s sleep problems. This finding suggests that parents’ HL may be a target for intervention to improve children’s sleep problems.

## Introduction

Given that the preschool age is a period of rapid functional and cognitive development, sleep in preschoolers is of critical importance [1, 2]. It has been reported that preschoolers in Asian countries have significantly later bedtime and shorter total sleep duration compared with other Western countries. Particularly among the Asian countries, Mindell et al. revealed that preschoolers in Japan have the shortest sleep time [3]. Sleep is important not only in terms of quantity, but also in terms of quality and habits among all generations, including preschoolers. The definition of “sleep problems” is all-encompassing and includes sleep quality, sleep quantity, and sleep habits [4] Sleep problems among preschoolers aged 4–5 years are associated with anxiety, [5] and among children aged 4–11 years, are associated with behavior problems and anxious/depressed mood [6]. Additionally, sleep problems among children aged 4–5 years predict poor health-related quality of life, behavior, language, and learning scores at age 6–7 years [7]. Of note, in a previous study, sleep problems at age 4 predicted adolescent depression and anxiety, and the strength of the relationship between sleep disorders and anxiety or depression increased with age [8]. Thus, sleep problems in preschoolers cause poor health outcomes in both the short-term and the long-term.

The construct of health literacy (HL) is crucial as a factor to maintain or improve health [9]. HL is defined as “personal skills that enable individuals to obtain, understand, and use information to make decisions and take actions that will have an impact on their health” [10]. The definition of HL has been modified and broadened, and it now consists of three subscales: functional HL, communicative HL, and critical HL. Low HL in adults has been associated with poor health outcomes such as obesity, [11] underweight, [12] and depression [13]. Further, low HL among parents has been reported to be associated with poor health outcomes for their children, such as poor body mass index (BMI), [14] low sleep duration, [15] and oral health deterioration [16]. In summary, parents’ HL is not only associated with their own health outcomes, but also their children’s health outcomes. Previous studies have suggested that parents play an important role in preschoolers’ sleep [17, 18]. Many previous studies have investigated the relationship between parents’ HL and children’s sleep duration [15, 18]; however, very few studies have focused on all aspects of sleep, including the regularity and quality of sleep. In addition, the relationship between parents’ HL and sleep problems among children is not clear. Therefore, we hypothesized that if parents have low HL, their children are more likely to have sleep problems. In this study, we investigate the true relationship between parents’ HL and sleep problems in children by comprehensively investigating parents’ HL, sleep problems in children, and the related factors.

Clarifying these relationships will serve as a breakthrough for improving children’s sleep disorders and, ultimately, their long-term health outcomes through interventions designed for their parents. The purpose of the present study was to assess the relationship between parents’ HL and sleep problems in their children.

## Method

### Participants

This was a cross-sectional multicenter study of children aged 3–6 years at two kindergartens, two nursery schools, and a Center for Early Childhood Education and Care in Chitose-city, Hokkaido, Japan. Questionnaires were distributed in February 2020, inviting preschool-aged children and their parents to participate in the study. Participation was completely anonymous and voluntary, and the final dataset was fixed after consultation with the heads of the multicenter facilities in October 2020.

In families with two or more children attending the same facility, only the oldest child was asked to participate in the study. Exclusion criteria included participants whose completed questionnaires had any missing values.

The study procedures were conducted in accordance with the Declaration of Helsinki and the Good Clinical Practice guidelines. This study was approved in advance by the Research Ethics Committee of Kobe University (approval number: 498–2). All participants were notified about their participation in this study and it was explained to them that they could withdraw their participation at any time.

### Demographic and health-related data

Data were self-reported by the parents for themselves and their children. The following is a list of the questionnaire items used for children: age (in months); sex (boy/girl); tooth decay (without decay, with decay); birth weight; number of siblings; breakfast (not every day, every day); prevalence of children’s after-school activities; sleep duration; and gaming and TV hours. BMI and the International Obesity Task Force (IOTF) [19] criteria were used to determine children’s weight status. BMI was calculated as the weight in kilograms divided by the height in meters squared. The IOTF classifies BMI as the norm to assess the prevalence of overweight and underweight conditions in children.

The following is a list of the questionnaire items used for parents: age (in years); sex (male, female); BMI; smoking and drinking habit; marital status (not married, married); education level (years); social economic status such as household income (≦6 million yen, > 6 million yen); sleep duration; PSQI (Pittsburgh Sleep Quality Index) [20]. PSQI subjectively measured sleep quality and assessed their children’s sleeping status.

### Health literacy in parents

The 14-item Health Literacy Scale (HLS-14) [21] developed in Japan was used to assess parents’ HL. A previous study has shown that the HLS-14 is an adequate instrument to assess comprehensive HL [21]. The questionnaire comprises 14 questions consisting of five items for functional HL, five items for communicative HL, and four items for critical HL. Each item is answered on a 5-point scale. The total score is calculated by adding the scores of all items. Higher scores indicate more comprehensive HL. The validity and reliability of this tool has already been demonstrated [22]. Participants were divided into two groups: high HL group (HLS-14 score > 50) and low HL group (HLS-14 score ≤ 50) [21]..

### Sleep problems in children

Sleep problems among children were assessed using the Japanese version of the Children’s Sleep Habits Questionnaire (CSHQ-J) [23]. It is one of the most widely used tools to comprehensively screen for children’s sleep problems. The CSHQ-J requires parents to retrospectively report their children’s sleep habits and behaviors. The scale includes 33 distinct items grouped into eight subscales with the following sleep domains: bedtime resistance, sleep onset delay, sleep duration, sleep anxiety, night wakings, parasomnias, sleep disordered breathing, and daytime sleepiness. Parents are asked to recall sleep behavior during a typical week and respond using the following options: “Usually” (5–7 times/week), “Sometimes” (2–4 times/week), and “Rarely” (0–1 times/week). Higher scores indicate more sleep problems.

### Statistical analysis

As mentioned above, we classified the study participants into two groups: a high HL group and a low HL group; this has been described in detail elsewhere [21]. Statistical analyses were conducted after confirming that the data were normally distributed using the Shapiro-Wilk test. The differences in the clinical characteristics between the two groups were determined using an unpaired *t*-test or chi-square test. Correlations between parents’ HL and the CSHQ-J scores were assessed using Pearson’s correlation coefficient. Multiple linear regression analysis was carried out to determine whether parents’ HL was associated with the children’s sleep states. The dependent variable was CSHQ-J or sleep duration in children, whereas the independent variable was parents’ HL. Multiple linear regression analyses were adjusted for characteristics of children and parents. We adjusted for children’s characteristics including their age, sex, IOTF, and number of siblings. At the same time, we adjusted for parents’ characteristics including their age, sex, BMI, educational status, household income, and smoking status. All statistical analyses were performed using EZR (Saitama Medical Center, Jichi Medical University, Saitama, Japan), which is a graphical user interface for R (The R Foundation for Statistical Computing, Vienna, Austria). Differences and correlations were considered significant when *p* < 0.05.

## Results

**Fig. 1:**
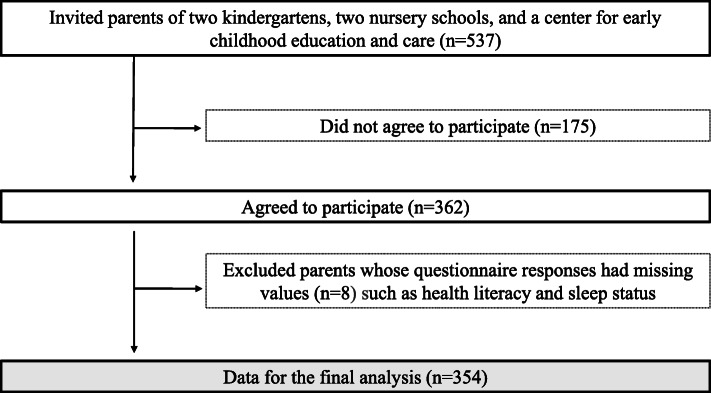
Participant flow during the study

Figure 1 shows the participant flow during this study. We distributed the questionnaires to 537 parents, of whom 362 (67.4%) agreed to participate in this study. Parents whose questionnaire responses had missing values (*n* = 8) were excluded. After the exclusions, the final sample for the analysis consisted of 354 parents and their children.

Table 1 shows the demographic differences between the low and high HL groups. The number of participants in the high HL group was 255 (72.0%) and that in the low HL group was 99 (28.0%). The average CSHQ-J score was significantly lower in the high HL group (45.3 ± 6.0 points) than in the low HL group (46.8 ± 5.9 points) (*p* = 0.043). In addition, parents’ BMI (*p* = 0.032) and years of education (*p* = 0.032) were higher in the high HL group than in the low HL group.

**Table 1:** Differences in characteristics between high health literacy and low health literacy groups

Characteristic	High HL (***n*** = 255)	Low HL (***n*** = 99)	***t or x***^***2***^	***P***-value
**Child**				
Age (months)	64.6 ± 10.3	63.3 ± 10.2	1.07	0.287
Sex (boys (%))	133 (52.2)	56 (56.6)	0.35	0.553
Teeth decay (non (%))	203 (79.6)	80 (80.8)	1.95	0.136
Birth weight (g)	2987.4 ± 445.1	3065.9 ± 399.9	−1.53	0.127
Number of siblings (n)	2.3 ± 0.8	2.2 ± 0.8	1.33	0.186
Breakfast (every day (%))	240 (94.1)	91 (91.9)	0.26	0.474
Lessons (%)	111 (43.5)	43 (43.4)	0	1.00
Sleep duration (hours)	10 ± 1.0	9.9 ± 1.1	1.21	0.228
Game (hours)	0.5 ± 0.8	0.5 ± 0.7	0.84	0.401
TV (hours)	2.1 ± 1.2	2.1 ± 1.2	0.22	0.826
BMI (kg/m^2^)	15.6 ± 1.5	15.6 ± 1.5	−0.13	0.896
IOTF (problem (%))	50 (19.6)	20 (20.2)	0	0.882
CSHQ-J (point)	45.3 ± 6.0	46.8 ± 5.9	−2.04	0.043
**Parent**				
Age (years)	36.1 ± 5.1	35.9 ± 5.8	0.41	0.685
Sex (men (%))	17 (6.7)	5 (5.1)	0.10	0.806
BMI (kg/m^2^)	21.1 ± 2.9	22.0 ± 4.0	−2.15	0.032
Smoking (%)	36 (14.1)	10 (10.1)	0.69	0.380
Drinking (everyday (%))	34 (13.3)	7 (7.1)	2.15	0.137
Material status (married (%))	238 (93.3)	96 (97.0)	2.01	0.699
Education level (years)	13.6 ± 1.7	13.1 ± 1.5	2.15	0.032
Income (> 6 million (%))	97 (38.0)	28 (28.3)	2.45	0.106
Sleep duration (hours)	6.8 ± 1.2	6.9 ± 1.2	−0.48	0.633
PSQI (alright (%))	165 (64.7)	53 (53.5)	3.26	0.066

Data are expressed as mean ± SD or number (percentage)

*HL* health literacy, *BMI* body mass index, *IOTF* International Obesity Task Force, *CSHQ-J* The Japanese version of Children’s Sleep Habits Questionnaire, *PSQI* Pittsburgh Sleep Quality Index

**Fig. 2:**
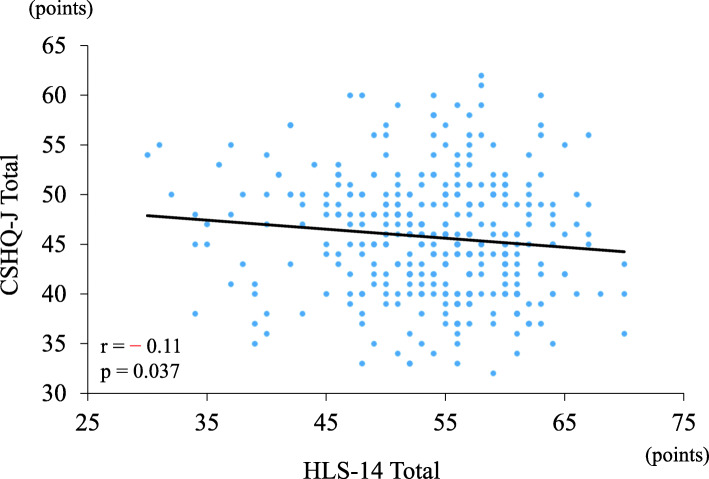
Correlation between parents’ health literacy and children’s sleep problems

The correlation between parents’ HL and children’s sleeping habits is illustrated in Fig. 2. There was a significantly negative correlation between parents’ HL and CSHQ-J (*p* = 0.0378, *r* = − 0.11).

Tables 2 and 3 show the results of the regression analyses. In the univariate regression analyses, CSHQ-J scores were statistically significantly associated with parents’ HL; nevertheless, children’s sleep duration did not show a statistically significant relationship with parents’ HL. In the multiple regression analysis, parents’ HL remained statistically significant in predicting CSHQ-J in children after adjustment for all other confounding factors such as children’s age, sex, IOTF, and number of siblings (*R*^2^ = 0.16, β = − 0.12; *p* = 0.03), but not the sleep duration of children (*R*^2^ = 0.17, β = 0.09; *p* = 0.08) (Model 1).

**Table 2:** Association of parents’ health literacy and sleeping problems in children according to multiple linear regression analyses

	Univariate model		Model 1 ^a^ *R*^*2*^ 0.16 Adjusted *R*^*2*^ 0.10		Model 2 ^b^ *R*^*2*^ 0.25 Adjusted *R*^*2*^ 0.22	
	*R*	*p* value	Standardized β	*p* value	Standardized β	*p* value
Dependent variable: CSHQ-J						
Parents’ health literacy	− 0.11	0.038	−0.12	0.03	−0.11	0.043

*CSHQ-J* The Japanese version of Children’s Sleep Habits Questionnaire, *R*^2^ Coefficient of determination, β Regression coefficient

^a^ Model 1 was adjusted for children’s age, sex, IOTF, and number of siblings

^b^ Model 2 was adjusted for Model 1 + parent’s age, sex, BMI, educational status, household income, and smoking status

**Table 3:** Association of parents’ health literacy and sleeping duration in children according to multiple linear regression analyses

	Univariate model		Model 1 ^a^ *R*^2^ 0.17 Adjusted *R*^*2*^ 0.15		Model 2 ^b^ *R*^2^ 0.24 Adjusted *R*^*2*^ 0.22	
	*r*	*p* value	Standardized β	*p* value	Standardized β	*p* value
Dependent variable: Sleeping duration in children						
Parents’ health literacy	0.08	0.16	0.09	0.08	0.04	0.44

*CSHQ-J* The Japanese version of Children’s Sleep Habits Questionnaire, *R*^*2*^ Coefficient of determination, β Regression coefficient

^a^ Model 1 was adjusted for children’s age, sex, IOTF, and number of siblings

^b^ Model 2 was adjusted for Model 1 + parent’s age, sex, BMI, educational status, household income, and smoking status

In addition to Model 2, parents’ HL remained statistically significant for predicting CSHQ-J in children after adjustment for parents’ age, sex, BMI, years of education, household income, and smoking status (*R*^2^ = 0.25, β = − 0.11; *p* = 0.043), but not the sleep duration of children (*R*^2^ = 0.24, β = 0.04; *p* = 0.44).

## Discussion

To our knowledge, this is the first study to assess the relationship between parents’ HL and children’s sleep problems. Consistent with our hypothesis, we found a statistically significant relationship between parents’ HL and sleep problems in children.

There are two possible reasons for the association between parents’ HL and sleep disorder in children. First, parents with low HL may be likely to create inappropriate living conditions and engage in parenting behaviors unfavorable to children’s sleep. A previous study suggested that low HL in parents is associated with suboptimal parenting practices and inadequate parenting behavior, such as putting a TV in the child’s bedroom [18]. Another study suggested that parents’ bedtime and the frequency of parents’ presence at the children’s bedtime are linked to children’s sleep problems [24]. Therefore, the behaviors of parents with low HL may play a role in their children’s sleep problems.

Second, parents with low HL may face mental health issues and parenting-related stress. For example, a study suggested that people with low HL were more likely to be depressed [25]. Another study suggested that parents of children with sleep problems were more likely to experience parenting stress, mental health issues, and depression [26]. Although studies investigating the mechanism between parents’ HL and children’s sleep problems are sparse, issues related to the mental health of parents with low HL may affect their children’s sleep problems.

In the present study, sleep duration was not measured directly but was reported by the parents. Previous studies have shown that children’s parent-measured sleep duration overestimated actual sleep duration by approximately 30 min [27]. Therefore, children’s sleep duration reported by their parents may not always be accurate and the results may be highly variable depending on the individual reporting.

According to Fig. 2, the parents’ HL scores were significantly but negatively correlated with the CSHQ-J scores, albeit the correlation was weak (*r* = − 0.11; weak: *r* < 0.4, moderate: 0.4 ≤ *r* < 0.6, strong: 0.6 ≤ *r* < 0.8). Moreover, parents’ HL was independently associated with CSHQ-J scores after adjusting for other confounding factors. This suggests that improving parents’ HL may lead to improvements in children’s sleep problems. In fact, there are many reports of HL interventions causing improvements in HL [28]. HL education might improve sleep quality but not sleep duration. More studies are required to examine the effectiveness of intervention for parents’ HL in the future. Further, although the present study dealt with the total CSHQ-J scores, investigating each item on the CSHQ-J may provide an opportunity to identify the causes of sleep problems in children.

There are several limitations to the present study. First, as this is a cross-sectional study, a causal relationship between parents’ HL and other factors cannot be concluded, and the mechanism by which parents’ HL affects sleeping problems in children is unclear. Second, a sampling bias may have occurred due to the small sample size and the fact that the data were collected in one particular area, that is, Chitose City, Hokkaido. Finally, it is possible that we did not collect enough information relevant to parents’ HL, and there are unknown confounding factors. This is evident from Table 2, which shows adjusted *R*^*2*^ = 0.25, meaning that only 25% of children’s sleeping problems were predicted by the factors we studied.

## Conclusion

The findings of this study suggest that parents’ HL independently affects the sleep problems of their children after adjusting for all other confounding factors. These results suggest that interventions targeting parents’ HL are needed to improve children’s sleep problems. Additionally, children’s sleep problems need to be examined in more detail.

## Data Availability

All relevant data are present within the paper. Furthermore, if additional information or permission is needed, for example, for use in a meta-analysis, it can be made available from the corresponding author for researchers who meet the criteria for access to confidential data.

## Abbreviations

CSHQ-J: The Japanese version of Children’s Sleep Habits Questionnaire
HLS-14: The 14-item Health Literacy Scale
